# Endogenous TDP-43, but not FUS, contributes to stress granule assembly via G3BP

**DOI:** 10.1186/1750-1326-7-54

**Published:** 2012-10-24

**Authors:** Anaïs Aulas, Stéphanie Stabile, Christine Vande Velde

**Affiliations:** 1 Centre d’excellence en neuromique de l’Université de Montréal, Centre de recherche du Centre Hospitalier de l’Université de Montréal (CRCHUM), Departments of Medicine and Biochemistry, Université de Montréal, 1560 rue Sherbrooke Est, Montréal, QC, H2L 4M1, Canada; 2 CHUM Research Center (CRCHUM), Université de Montréal, 1560 rue Sherbrooke Est, Montréal, H2L 4M1, Canada

**Keywords:** TDP-43, Stress granules, FUS/TLS, G3BP, TIA-1, ALS, Cell death, Oxidative stress

## Abstract

Amyotrophic lateral sclerosis (ALS) is a fatal neurodegenerative disease characterized by the selective loss of upper and lower motor neurons, a cell type that is intrinsically more vulnerable than other cell types to exogenous stress. The interplay between genetic susceptibility and environmental exposures to toxins has long been thought to be relevant to ALS. One cellular mechanism to overcome stress is the formation of small dense cytoplasmic domains called stress granules (SG) which contain translationally arrested mRNAs. TDP-43 (encoded by *TARDBP*) is an ALS-causative gene that we have previously implicated in the regulation of the core stress granule proteins G3BP and TIA-1. TIA-1 and G3BP localize to SG under nearly all stress conditions and are considered essential to SG formation. Here, we report that TDP-43 is required for proper SG dynamics, especially SG assembly as marked by the secondary aggregation of TIA-1. We also show that SG assembly, but not initiation, requires G3BP. Furthermore, G3BP can rescue defective SG assembly in cells depleted of endogenous TDP-43. We also demonstrate that endogenous TDP-43 and FUS do not have overlapping functions in this cellular process as SG initiation and assembly occur normally in the absence of FUS. Lastly, we observe that SG assembly is a contributing factor in the survival of neuronal-like cells responding to acute oxidative stress. These data raise the possibility that disruptions of normal stress granule dynamics by loss of nuclear TDP-43 function may contribute to neuronal vulnerability in ALS.

## Background

Amyotrophic Lateral Sclerosis (ALS) is a late-onset neurodegenerative disease characterized by the selective loss of upper and lower motor neurons. It is inevitably fatal with patients experiencing a progressive and rapid paralysis owing to a relentless denervation of nearly all muscles. ALS can be either inherited with 10% of cases having a familial history, or be sporadic where etiology remains unknown but exposure to environmental stresses are strongly suspected to be relevant [1]. Of the familial cases, ~4% are due to mutations in *TARDBP*, encoding TAR DNA Binding Protein (TDP-43) [2,3]. Interestingly, a proportion of sporadic ALS cases have also been attributed to TDP-43 mutations [2,3]. TDP-43 is a heterogeneous nuclear ribonucleoprotein (hnRNP) and is reported to function in multiple aspects of RNA metabolism including transcription, splicing, and stabilization [4]. Mutations in a second hnRNP, FUS/TLS (Fused in sarcoma/translocated in sarcoma), are also causative for ALS [5,6]. TDP-43 and FUS are expected to function similarly in RNA metabolism [4].

Mammalian cells possess a variety of mechanisms to mediate cellular recovery following stress exposure. One process is the transient formation of stress granules (SG), small cytoplasmic inclusions (≤ 5 μm) that rapidly form following exposure to chemical, viral or thermal stress [7,8]. SG contain several RNA-binding proteins, stalled pre-initiation complexes and translationally arrested mRNAs. They are believed to function as sites for mRNA storage and sorting, permitting a rapid re-integration of these stalled pre-initiation complexes once the stress has been cleared [8]. SG formation is a highly regulated process that is initiated by the self-oligomerization of the core SG proteins G3BP (RasGAP SH3 domain binding protein 1) and TIA-1 (T-cell intracellular antigen) within 15-20 min of stress exposure [9,10]. In the case of acute oxidative stress, several small SG fuse into fewer but larger SG over the next 60 min. This secondary aggregation is referred to as SG assembly [8]. By 2 hours post-stress exposure, SG disassemble and cellular processes return to normal [11,12]. Thus, SG are a transient mechanism to halt general protein translation and give translational priority to specific transcripts that are necessary for the cell to overcome the encountered stress [11].

Efficient SG kinetics are considered imperative to cell survival following exposure to a variety of physiological and environmental stresses [13-15]. Impaired SG signalling as a contributing factor in neurodegenerative diseases has been previously documented. Specifically, depletion or mutation of FMRP, the gene responsible for the neurological Fragile X syndrome, induces a severe impairment in SG dynamics [16]. A variant in SMN responsible for the motor neuron disease spinal muscular atrophy (SMA) fails to be recruited normally to SG in SMA patient cells [17]. Ataxin-2, mutated in spinocerebellar ataxia type 2 and recently linked as a susceptibility gene for ALS, localizes to and regulates SG formation [18-22].

TDP-43 and FUS both localize to SG in response to oxidative, thermal, osmotic, and proteasomal stress [12,23-28]. TDP-43 is localized to cytoplasmic inclusions in post-mortem tissue from ALS patients and since some of these inclusions are also positive for SG markers, some have suggested that deregulation of SG may drive inclusion formation [23,29,30]. However, in a cell-based assay, very few TDP-43 positive SG actually proceed to aggregate formation [30]. Thus, it is important to remember that the other striking pathological feature observed in patient motor neurons is the depletion of the nuclear pool of TDP-43. Whether TDP-43 mutations are a gain or loss of function (or both) is currently a matter of debate [4,31,32]. However, it has recently been proposed that TDP-43 pathogenesis might arise from a loss of nuclear TDP-43 function (and/or gain of cytoplasmic function) [31]. In fact, data from yeast to rodents indicates that TDP-43 mediated toxicity correlates better with a loss of normal TDP-43 function than with the presence/generation of TDP-43 cytoplasmic aggregates [31,33]. TDP-43 is primarily localized to the nucleus. Thus, in order to mimic the nuclear depletion that is universally observed in neurons of patient brains and spinal cords [34,35], we have employed siRNA-specific depletion of TDP-43 as a model of loss of nuclear function. Using this system, we have previously demonstrated that TDP-43 modulates the expression of the core SG factors G3BP and TIA-1 [12]. Here, we have addressed the role of endogenous TDP-43 in SG formation and assembly in response to oxidative stress documenting that endogenous TDP-43 and FUS do not have overlapping functions in this process. Furthermore, we report that the TDP-43 target gene G3BP is required for SG assembly but not initiation. Surprisingly, we demonstrate that blocked SG assembly (secondary TIA-1 aggregation) has a differential effect on the survival of neuronal and non-neuronal cells responding to acute oxidative stress exposure.

## Results

### Endogenous TDP-43 regulates SG assembly as marked by secondary TIA-1 aggregation

We have previously demonstrated that TDP-43 depletion leads to a defect in the timing of SG formation in response to acute oxidative stress induced by sodium arsenite (SA) treatment [12]. In addition, we reported that SG in TDP-43 depleted cells assayed immediately after the stress were 43% smaller than control cells [12], but the number of SG per cell is comparable to siControl cells (73.4 ± 3.6 vs. 82.2 ± 6.4, p = 0.15). These data suggest a potential role for TDP-43 in SG dynamics. It is well documented that following initial SG formation, SG slowly reduce in number but each individual SG increases in size before resolving. This SG size increase is attributed to the fusion of multiple TIA-1 oligomers and is referred to as SG assembly [8]. However, the mechanism(s) which mediate this step is unclear.

**Figure 1:**
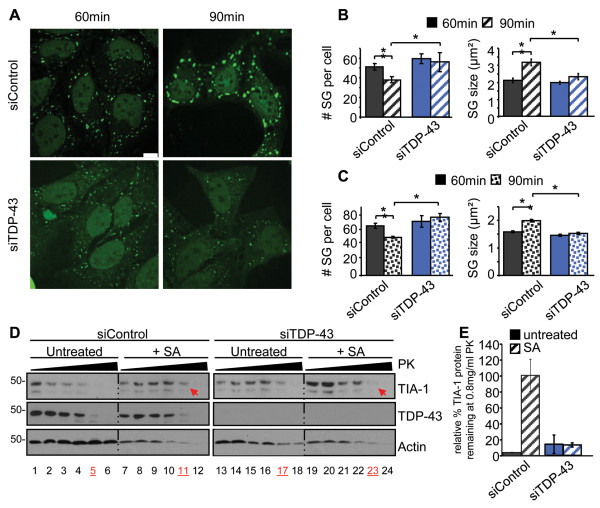
**TIA-1 secondary aggregation is impaired by TDP-43 depletion. (A-D)** Cells where transfected with control or TDP-43 siRNA for 72 hr and subsequently treated with SA. **(A)** Representative images of SG in HeLa cells at 60 and 90 min. Scale bar, 10 μm. **(B & C)** Quantification of SG number and size by semi-automatic analysis using ImageJ at 60 and 90 min after SA treatment in **(B)** HeLa and **(C)** SK-N-SH cells. The means of 3 independent experiments ± SEM are plotted. * p<0.05. **(D)** siRNA transfected HeLa cells were treated with or without SA and collected 1 hr post-SA. Cytoplasmic extracts were digested with 0, 0.1, 0.2, 0.4, 0.8 or 1.6 mg/ml Proteinase K and assayed by immunoblot. TIA-1 is more protease-sensitive when TDP-43 is absent (arrows). Data is representative of 3 independent experiments. **(E)** Quantification of remaining TIA-1 protein following treatment with 0. 8 mg/ml Proteinase K, expressed relative to untreated samples.

Building on our previous published data in which we validated two independent TDP-43 specific siRNAs, we selected one of these siRNAs for transfection into HeLa cells and then subsequently stressed the cells for 30 min with 0.5 mM SA followed by recovery with fresh media. Cells were subsequently fixed at 60 and 90 min and SG were visualized with TIA-1 labelling (Figure 1A). In HeLa cells transfected with control siRNA (siControl), we observed an average of 84 ± 5 SG per cell with an average size of 1.9 ± 0.1 μm^2^ at 60 min, followed by a reduction in SG number (60 ± 6 SG, p = 0.005) and an increase in SG size (to 3.0 ± 0.1 μm^2^, p = 0.009) at 90 min (Figure 1B). This simultaneous decrease in SG number and increased SG size is due to the secondary aggregation of TIA-1 oligomers and is referred to as SG assembly [8]. In cells depleted of endogenous TDP-43, SG assembly is blocked such that SG number and size remains unchanged at both time points examined (Figure 1B). A second TDP-43 targeted siRNA also yielded the same results (Additional file 1: Figure S1). Moreover, this blockade in SG assembly was also true in SK-N-SH cells, a neuronal-like cell line (Figure 1C).

To further evaluate whether TIA-1 oligomerization was disrupted in SG assembly, we performed a protease sensitivity assay. Briefly, SG assembly is reflected as increased TIA-1 aggregation and thus increased protease resistance [9,36]. Cells transfected with TDP-43 or control siRNA were stressed with SA and then permitted to recover for 60 min before cytoplasmic fractions were subject to a titration of Proteinase K (PK) and blotted for TIA-1. In siControl cells, we observed an increased resistance of TIA-1 to proteolysis between stressed and unstressed cells (Figure 1D, *compare lanes 5 and 11*; Figure 1E). In contrast, TIA-1 protease-sensitivity in SA-treated TDP-43 depleted cells was comparable to untreated cells, indicating decreased TIA-1 aggregation and thus impaired SG assembly (Figure 1D, *compare lanes 11 and 23, red arrows*; Figure 1E). Collectively, these experiments demonstrate that endogenous TDP-43 is required for normal SG assembly.

### The TDP-43 target G3BP is required for SG assembly but not initiation

**Figure 2:**
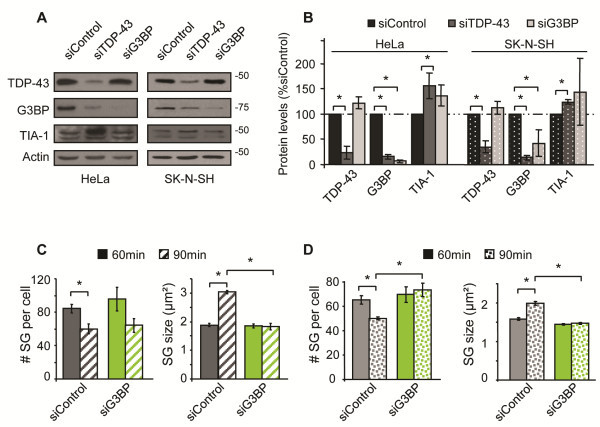
**The TDP-43 target gene G3BP is required for SG assembly.(A)** Western blot to confirm efficacy of indicated siRNAs in HeLa (left panel) and in SK-N-SH (right panel) cells. Actin serves as a loading control. **(B)** Quantification of protein levels demonstrating that G3BP does not regulate TDP-43 in HeLa (left panel) nor in SK-N-SH (right panel). **(C & D)** Quantification of individual SG size and number in siRNA treated cells, at 60 and 90 min after SA treatment in **(C)** HeLa cells and **(D)** SK-N-SH cells. The means of 3 independent experiments ± SEM are plotted. * p < 0.05. siControl in Figure 2C is the same presented in Figure 4A,B. siControl in Figure 2D is the same presented in Figure 1C.

We have previously reported that G3BP is down-regulated in cells depleted of TDP-43 via two independent siRNAs [12]. We hypothesized that the SG assembly defect observed in TDP-43 depleted cells could be attributed to decreased G3BP expression. To address this, we reduced G3BP levels using a gene-specific siRNA to levels comparable to cells treated with TDP-43 siRNA (remaining G3BP protein in siG3BP vs. siTDP-43 in HeLa cells: p = 0.08; in SK-N-SH: p = 0.32; Figure 2A, B). Importantly, we noted that G3BP depletion does not impact TDP-43 expression (HeLa: p = 0.071, SK-N-SH: p = 0.21; Figure 2A, B). G3BP is considered essential for SG initiation based on studies where overexpression of G3BP is sufficient to nucleate SG [10]. However, unlike TIA-1, which is required for SG initiation, the impact of G3BP on SG initiation by either siRNA or genetic deletion has not been reported. To define if G3BP is necessary for SG initiation at endogenous expression levels, we counted the number of SG per cell and measured SG size in G3BP-depleted conditions immediately following a 30 min SA exposure. We observed that the number of SG per cell was equivalent in siControl and siG3BP cells (113 ± 3 vs. 117 ± 11 SG per cell, p = 0.373, Additional file 2: Figure S2). Moreover, we did not detect a difference in initial SG size (1.7 ± 0.1 vs. 1.8 ± 0.1 μm^2^, p = 0.398, Additional file 2: Figure S2). Since SG composition can vary between cell types, we also evaluated SK-N-SH cells and observed strikingly similar results with respect to SG number (55 ± 8 vs. 63 ± 13 SG per cell, p = 0.332) and size (1.43 ± 0.01 vs. 1.42 ± 0.03 μm^2^, p = 0.366). Together, these data indicate G3BP is a non-essential factor in the initiation of SG in response to acute oxidative stress induced by SA.

To evaluate the role of G3BP in SG assembly, we quantified the number of SG per cell as well as individual SG size at 60 and 90 min after SA treatment. Visual inspection of SG by confocal suggested a difference in SG number and size in siG3BP cells compared to controls. Quantification of individual SG in these cells revealed that the number and the size of SG in siG3BP HeLa cells is comparable to siControl cells at 60 min (p = 0.243 and p = 0.400, respectively; Figure 2C). In contrast, while average SG size increases 1.6-fold in siControl cells (p = 0.004) as expected, siG3BP cells remain constant at 1.9 ± 0.1 μm^2^. We obtained similar results with a second siRNA for G3BP (Additional file 1: Figure S1) and in SK-N-SH cells (Figure 2D) indicating that the requirement of G3BP for SG assembly is not a cell-type specific event. Taken together, our data demonstrate G3BP as an important factor in SG assembly, but not SG initiation as previously suggested.

### G3BP rescues the defect in SG assembly observed in TDP-43 depleted cells

**Figure 3:**
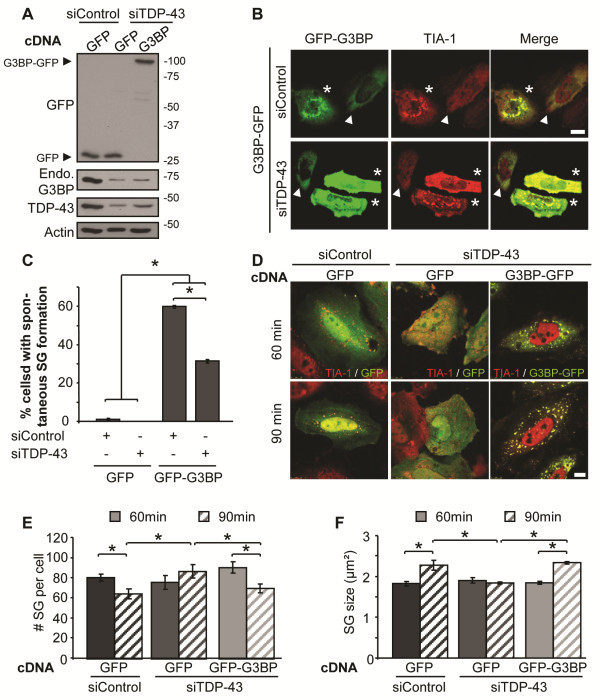
**Blocked SG assembly in TDP-43 depleted cells is rescued by G3BP-GFP. (A - F)** SG formation and resolution were assessed in HeLa cells transfected with control or TDP- 43 siRNA for 48 hr, transfected with GFP or G3BP-GFP for 24 hr and subsequently treated with SA. Cover slips were collected before SA treatment **(A – C)** and at 60 and 90 min after stress **(D - F)**. **(A)** Western blot confirming expression of transfected plasmids. Actin serves as a loading control. **(B)** Representative micrographs of quantified cells with spontaneous SG formation. Asterisks and arrows indicate cells expressing G3BP-GFP with or without spontaneously forming SG, respectively. Scale bar, 20 μm. **(C)** Quantification of cells with spontaneous SG formation (SG formation in the absence of stress). Cells were scored as SG positive when they had at least two TIA-1 foci of a minimal size of 0.75 μm^2^. **(D)** Representative images quantified for SG number and size by semi-automatic analysis using ImageJ. Scale bar, 10 μm. **(E & F)** Number and size of SG were quantified at 60 and 90 min after SA treatment by semi-automatic analysis using ImageJ. The means of 3 independent experiments ± SEM are plotted. * p < 0.05.

Our previous work indicated that TDP-43 depletion using two independent siRNAs resulted in a robust and reproducible decrease in G3BP expression [12]. To investigate whether the defect in SG assembly observed in TDP-43 depleted cells could be attributed to G3BP, we transiently expressed GFP-tagged G3BP in TDP-43 depleted cells, at a level comparable to endogenous G3BP expression (Figure 3A). Using our conditions, where we transfected 0.3 μg of G3BP-GFP cDNA for 24 hr, we detected cells of both high and low expression by immunofluorescence. Consistent with a previous report, G3BP overexpression at high levels induces the spontaneous formation of SG [10] (Figure 3B, *asterisks*) such that 60 ± 0.4% of transfected cells formed SG in the absence of SA treatment (Figure 3C). Interestingly, we noted that the depletion of TDP-43 resulted in a 1.9-fold reduction in the number of cells with spontaneous SG formation suggesting that endogenous TDP-43 participates in this process (p = 0.0006).

In order to address our initial hypothesis that G3BP mediates SG assembly in TDP-43 depleted cells, we selected transfectants with low G3BP-GFP expression for further analysis. Low expression was defined via measurement of the GFP signal in individual cells. Cells having an average pixel intensity below 60 arbitrary fluorescent units were uniquely included in the subsequent quantification. This population of low-expressing G3BP-GFP cells do not exhibit spontaneous SG formation (Figure 3B, *arrowheads*). As expected, cells co- transfected with control siRNA and control GFP cDNA undergo normal SG assembly while siTDP-43 cells transfected with GFP cDNA demonstrate a defect in SG assembly (Figure 3D-F). The low expression of G3BP-GFP in TDP-43 depleted cells restored the number and size of SG at 90 min to levels equivalent to controls, indicating a restoration of SG assembly (Figure 3E, F). Thus, the defect in SG assembly in TDP-43 depleted cells can be rescued by G3BP expression. These data indicate that G3BP functions downstream of TDP-43 in SG assembly.

### SG formation and assembly does not require endogenous FUS

**Figure 4:**
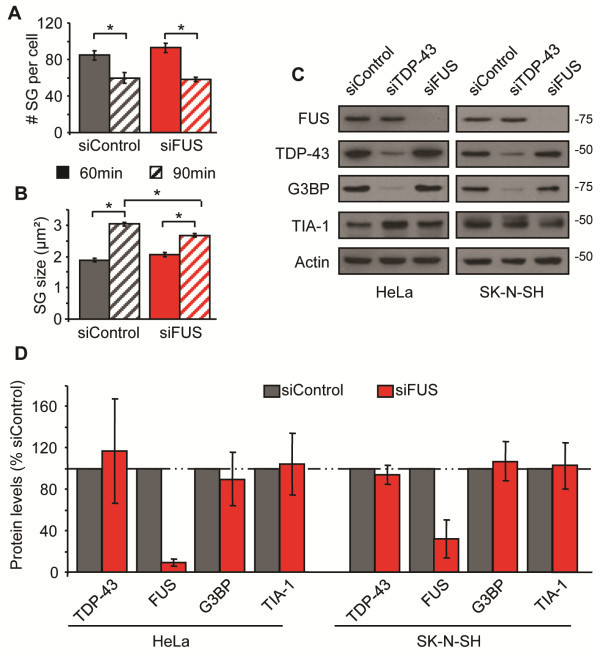
**Endogenous FUS does not function in SG assembly in response to sodium arsenite. (A - E)** SG were assessed in HeLa cells transfected with control or FUS siRNA for 72 hr and subsequently treated with SA. **(A & B)** Quantification of individual SG size and number of SG per cell. **(C & D)** Western blots and quantification of protein levels demonstrating that FUS does not regulate G3BP, TIA-1 or TDP-43, in HeLa cells (left panel) nor in SK-N-SH cells (right panel). Actin serves as a loading control. The means of 3 independent experiments ± SEM are plotted. * p < 0.05

There have been several reports that a second ALS-causing gene FUS/TLS, which is an hnRNP like TDP-43, localizes to SG in response to oxidative stress [26-28]. All of these studies demonstrated SG localization of over-expressed FUS protein, but do not evaluate the contribution of endogenous FUS to this process. Since there is suggestion that TDP-43 and FUS participate in similar biological processes [37-40], we investigated the potential role of endogenous FUS in SG dynamics in response to oxidative stress using siRNA depletion in HeLa cells. We did not observe any difference in the number of cells able to form TIA-1 labelled SG in response to SA treatment at any time point examined (data not shown). Furthermore, SG initiation (number and size at 30 min) was undisturbed in cells depleted of FUS (Additional file 2: Figure S2). Similarly, SG assembly proceeded normally as SG number and size were similar between siControl and siFUS cells at 60 and 90 min post-stress (Figure 4A, B). These data were confirmed with a second siRNA specific for FUS (Additional file 1: Figure S1). Since TDP-43 depletion can alter the expression of TIA-1 and G3BP [12], we also evaluated the levels of these proteins in siFUS cells. FUS depletion (92% in HeLa and 68% in SK-N-SH) did not impact TIA-1 or G3BP protein expression in HeLa or SK-N-SH cells (Figure 4C, D). We also noted that FUS depletion does not impact the expression of endogenous TDP-43 (Figure 4C, D) nor does it impact TDP-43 localization to SG (data not shown). We conclude that endogenous wild type FUS does not participate in SG initiation or assembly in response to acute oxidative stress.

### TDP-43 mediates cellular vulnerability to oxidative stress in neuronal-like cells

**Figure 5:**
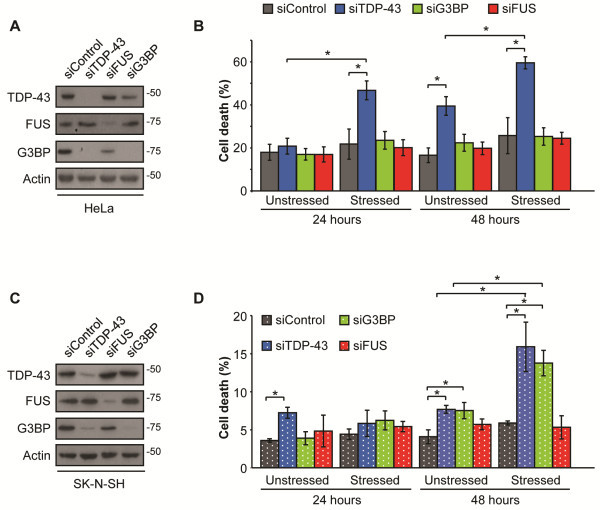
**Neuronal-like cells have increased vulnerability to blocked SG assembly. (A & B)** HeLa and **(C & D)** SK-N-SH cells treated with the indicated siRNAs for 48 hr and then exposed to SA for 30 min. **(A & C)** Western blots confirming siRNA transfections in **(A)** HeLa and **(D)** SK-N-SH cells. Actin serves as a loading control. **(B & D)** Cell death determined by trypan blue exclusion at the indicated times following SA treatment in **(B)** HeLa and **(D)** SK-N-SH cells. The mean percentage of cell death is presented ± SEM of 3 independents experiments. * p < 0.05

SG comprise one arm of an integrated cellular stress response. Previous data indicate that a failure in SG dynamics (meaning formation, assembly, or disassembly) contributes to poor cellular recovery to acute stress [13-15]. We have previously shown that TDP-43 contributes to cell survival 24 hr after stress exposure [12]. To determine if this was a short- or long-term requirement and whether this was exclusive to TDP-43, we extended these studies such that cells transfected with specific siRNAs for 48 hr were stressed (SA, 30 min) and then assessed for cell viability at 24 and 48 hr by trypan blue exclusion. In all cases, robust depletion of TDP-43, G3BP, and FUS was achieved by the respective siRNAs (Figure 5A, C). In the case of TDP-43 depleted HeLa cells, we noted a 1.9-fold increase (p = 0.01) in cell death in unstressed conditions at 48 hr while no significant differences in viability were detected in siG3BP or siFUS cells (Figure 5B). When challenged with SA treatment, siTDP-43 cells fared worse than siControl cells 24 hr later and cell death was further increased to 2.3-times siControl cells at 48 hr (24 hr: p = 0.02; 48 hr: p = 0.01, Figure 5B). Depletion of FUS had no impact on cell viability at any time point, consistent with our data that endogenous FUS is not required for a SG response. Surprisingly, depletion of G3BP did not impact cellular recovery to oxidative stress in HeLa cells (Figure 5B). However, additional experiments in the neuronal-like cell line SK-N-SH revealed similarly increased cell vulnerability 48 hr post-SA treatment in both TDP-43 and G3BP depleted cells (siTDP-43: 2.3-fold, p = 0.019; siG3BP: 2.6-fold, p = 0.005) (Figure 5D). In contrast, as in HeLa cells, FUS depletion had no effect on survival in these neuronal-like cells. Thus, neuronal-like cells seem to be more vulnerable to a defect in SG assembly when either TDP-43 or G3BP is depleted. In both lines, cells transfected with control siRNA exhibited a background level of cell death that was constant throughout the experiments.

### TIA-1 overexpression enhances cell toxicity of HeLa cells

**Figure 6:**
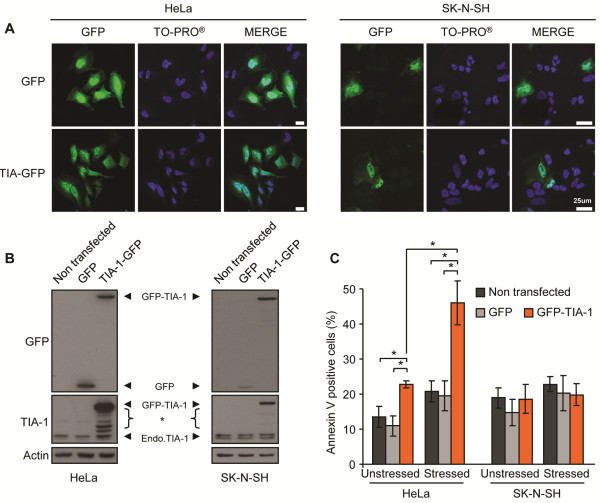
**HeLa cells have increased cell sensitivity to sodium arsenite in the presence of modestly elevated GFP-TIA-1 expression. (A–C)** HeLa (left panels) and SK-N-SH (right panels) cells were transfected with GFP or GFP-TIA-1 for 48 hr. (**A**) Representative images of HeLa (left panels) and SK-N-SH (right panels) cells transfected with GFP or GFP-TIA-1 for 48 hr and then stained with TO-PRO3 to label nuclei. **(B)** Western blot confirming TIA-1 expression. Asterisk (*) indicates non-specific bands from TIA-1 antibody. **(C)** Cell viability was assessed by Annexin V labelling in HeLa and SK-N-SH. The means of 3 independent experiments ± SEM are plotted. * p<0.05

TDP-43 depleted cells have not only a down-regulation of G3BP but also a 30% up-regulation of TIA-1 protein levels (and 2.6-fold increase in TIA-1 transcripts) [12] that is not observed in G3BP depleted cells (Figure 2A). Since G3BP siRNA failed to recapitulate the same level of toxicity as TDP-43 depletion in HeLa cells, we wondered if there was a differential sensitivity to elevated TIA-1 expression between cell types. To address this, we transiently transfected cells with a low concentration of GFP-tagged TIA-1 for 24 hr (Figure 6A, B). At 24 hr post-transfection, we observed that cells with high GFP-TIA-1 expression formed puncta resembling SG in the absence of exogenous stress, in agreement with a previous report [9]. (However, TIA-1 aggregates were not detected at 48 hr, data not shown.) Cells were then briefly stressed with SA (30 min) and the media was replaced so as to permit cellular recovery for 24 hr. In order to avoid inducing non-specific cell death attributed to supraexpression of TIA-1, we used a very low concentration of cDNA in these experiments. This resulted in a low transfection efficiency. Thus, in order to selectively assess cell death in GFP^+^ transfectants (and thus exclude non-transfectants), we employed a flow cytometry method to assess cell death via Annexin V labelling specifically in GFP-expressing cells. By this method, we observed that GFP-TIA-1 transfected HeLa cells have a 2.3-fold increased toxicity following SA treatment (p = 0.01, Figure 6C). Also, we note that in unstressed/basal conditions, GFP-TIA-1 transfected cells have nearly twice the toxicity as control GFP transfected cells (p = 0.005, Figure 6C). Note, while TIA-1 has been previously described as cytotoxic, the toxicity observed here is independent of the level of TIA-1 overexpression as demonstrated by independently gating populations of high and low GFP-TIA-1 expression (Additional file 3: Figure S3). In contrast, control GFP transfected cells were comparable to untransfected cells in both basal and stressed conditions. When we performed the same experiment in SK-N-SH cells, we noted that TIA-1 over-expression did not sensitize cells to SA (Figure 6C). Note, the apparent difference in TIA-1 expression detected by immunoblot likely arises from a reduced transfection efficiency of SK-N-SH cells (compare images in Figure 6A), while FACS analysis was restricted to cells expressing either GFP or GFP-tagged fusion protein. Taken together, these data suggest that low-level overexpression of TIA-1 can be toxic to certain cell types and this toxicity can be exacerbated by the addition of an exogenous stress.

## Discussion

We previously reported that cells depleted of endogenous TDP-43 formed smaller and less well-defined SG in response to oxidative stress. In addition, we reported that the reduction of endogenous TDP-43 levels induced an up-regulation of TIA-1 and a down-regulation of G3BP [12]. G3BP and TIA-1 are both considered to be SG nucleating proteins based on studies where overexpression of either molecule is sufficient to induce SG formation in the absence of stress [9,10]. While the requirement for TIA-1 in SG initiation has been further reinforced by the use of TIA-1 null cells [9], a similar experiment has not been reported for G3BP. Using siRNA-specific depletion of G3BP, we found that SG initiation defined by primary TIA-1 aggregation was undisturbed. We conclude that G3BP is not an essential SG initiating factor. In contrast, SG assembly defined by the secondary aggregation of TIA-1 resulting in fewer but larger SG, was completely blocked by the reduction of G3BP. This effect was nearly identical to that observed in TDP-43 depleted cells. Moreover, the blockade in SG assembly in TDP-43 depleted cells could be restored by the addition of exogenous G3BP. Our data indicate that TDP-43 regulation of G3BP controls SG assembly, an important step in SG dynamics. Interestingly, a large-scale analysis of TDP-43 target mRNAs has identified TDP-43 as binding to G3BP mRNA at the 3’-UTR [41]. Our own experiments in HeLa cells using immunoprecipitation of TDP-43 followed by RT-PCR for G3BP transcripts, confirm that TDP-43 can indeed bind G3BP mRNA in at least one of the cell types used here (unpublished data). The exact mechanism(s) by which TDP-43 regulates G3BP expression is the focus of future investigations. Interestingly however, we note that our data is strikingly similar to observations made in poliovirus infection where G3BP is cleaved by the virus-encoded 3C protease [42]. In this context, G3BP cleavage effectively inactivates G3BP, yielding smaller SG compared to oxidative stress exposure [42,43]. It is poignant to point out that poliovirus, which selectively attacks motor neurons, can result in a post-infection syndrome characterized by progressive muscle weakness and paralysis in rare cases. Lastly, it is noteworthy that G3BP-null mice are embryonically lethal, with animals largely developing normally with the exception of widespread neuronal cell death in various brain regions (spinal cord was not examined) [44]. Collectively, these data raise the possibility that SG dynamics (and the G3BP axis) are an essential aspect to motor neuron survival. Future studies include validation of this hypothesis in primary motor neurons.

Our previous work demonstrated that SG were morphologically irregular and significantly smaller in cells depleted of TDP-43 immediately following acute oxidative stress [12]. We add here that these changes occur while the number of SG per cell remains constant. Collectively, these data suggest that TDP-43 can also contribute to SG initiation. Whether the effect is direct or indirect remains to be determined. As is the case for G3BP and TIA-1, it is possible that TDP-43 controls the expression of other SG residents essential to the process. Further study of this aspect is required to identify a TDP-43-regulated SG initiation factor(s) and/or determine if it is TDP-43 itself. While it is tempting to suggest that it is G3BP which mediates this step, we note that initial SG number and size is normal in G3BP depleted cells. Clearly, endogenous TDP-43 contributes to the regulation of SG dynamics at more than one level.

The function of SG is proposed to be the storage and sorting of mRNA and SG are considered to be an important component of cell survival following stress exposure [8]. Our experiments indicate that endogenous TDP-43 is required to overcome acute oxidative stress in both HeLa and the neuronal-like cell line SK-N-SH. In contrast, endogenous FUS was not required in either cell line. Taken together, we propose that TDP-43 toxicity in ALS favours a loss of protective function while FUS mutations do not. In fact, given the number of studies using cell models with overexpressed FUS cDNAs, we propose that FUS mutations are gain-of-function since overexpression seems to encourage SG localization, with mutant FUS having a stronger localization than wild type [26-28]. In addition, it has been reported that mutant FUS is recruited to SG more rapidly than wild type FUS suggesting that FUS mutation causes a possible imbalance in normal SG dynamics [27]. While this hypothesis may prove to be true, we find it interesting that depletion of endogenous FUS had no impact on SG initiation and assembly in the context of acute oxidative stress.

Since TDP-43 and G3BP depletion yields the identical effect on SG dynamics, we expected to observe the same effect in our cell survival assay. To our surprise, our hypothesis was true only in neuronal-like SK-N-SH cells. That SG composition and/or kinetics are not identical in different cell types has been previously reported [7,45]. Thus, while it is possible that HeLa cells have evolved other means to avoid cell death in the absence of normal SG kinetics, we feel that this data supports our hypothesis that the proper control of SG dynamics is essential to neuronal survival. Thus, the disruption of this mechanism could be an underlying element of motor neuron vulnerability in ALS. However, given that the current data is limited to transformed cell lines and acute oxidative stress, future experiments in primary cultures exploring other ALS-relevant stress agents will be required to fully evaluate this hypothesis.

TDP-43 depletion also leads to increased TIA-1 expression. While TIA-1 overexpression results in spontaneous SG formation, SG formation in the absence of SA was never observed in TDP-43 depleted cells. Thus, we wondered what may be the impact of moderately elevated TIA-1 levels. TIA-1 is a strong translational repressor [46] and is reported to induce apoptotic DNA fragmentation in thymocytes [47]. In our hands, ectopic TIA-1 expression induced spontaneous SG at 24 hr post-transfection. However, these inclusions were no longer observed at 48 hr, either due to cell death or spontaneous resolution. Moreover, we also detected a consistent 2-fold increase in cell death at 48 hr post-transfection. Surprisingly, the expression of TIA-1 exacerbated HeLa cell death 2.3-fold in response to an acute exposure to SA. We speculate that the modest up-regulation of TIA-1 induced by TDP-43 depletion (which does not yield visible aggregates), leads to a protracted translational arrest that contributes to increased cellular vulnerability. SK-N-SH cells were resistant to exogenous TIA-1 expression. Motor neurons, the vulnerable cell type in ALS, have very particular mRNA storage and protein translation mechanisms at the synapse [48]. In the context of ALS, we speculate that the reduction of functional TDP-43 levels (possibly via nuclear depletion) leads to a deregulation of normal SG kinetics and altered molecular control of key components relevant to cell survival. This contributes to a prolonged translational arrest and increased cell vulnerability. While our data implies a functional significance to the size/morphological changes associated with SG assembly, future work is needed to more fully examine the enigma that is “SG function” and the impact of arrested translation. However, it is noteworthy that increased SG size is widely viewed as a prerequisite to proposed SG function in mRNA triage [8].

Mutations in both TDP-43 and FUS are causative for ALS. In addition, both are RNA binding proteins that localize to SG in response to thermal or oxidative stress [12,23,25-28]. Many have expected that TDP-43 and FUS would participate in the same cellular pathways and that mutations in each would yield similar effects. However, we demonstrate here that the role for TDP-43 in regulating SG dynamics appears to be specific since depletion of endogenous FUS does not impact SG initiation or assembly. Moreover, at the molecular level, while TDP-43 and FUS have been reported to cooperatively regulate HDAC6 [39], we note here that reduction of FUS levels does not impact G3BP or TIA-1 protein levels in either of the cell types tested. In addition, in our experiments FUS depletion does not impact TDP-43 levels, nor does TDP-43 appear to regulate FUS, in contrast to a previous report [41]. This difference may be due to cell type since this study assayed whole mouse brain, while our study has examined cultured cell lines.

## Conclusions

SG are a transient mechanism to manage cell stress. To neurons, a non-renewable cell type, SG and other cellular stress response mechanisms are anticipated to be extremely important to their long-term survival [31,49]. We demonstrate that endogenous TDP-43, via G3BP, plays a role in SG dynamics that is independent of endogenous FUS. This supports our view that TDP-43 and FUS are not inter-changeable in all biological processes. We also report that SG assembly plays a more significant role in cell recovery following exposure to exogenous stress in neuronal-like cells, compared to non-neuronal cells. For future consideration, we hypothesize that this inherent and subtle difference in SG dynamics may be an underlying feature of the selective cellular vulnerability in ALS (and possibly other neurodegenerative diseases). Further studies using ALS relevant cell types and disease-linked stressors are warranted.

## Methods

### Cell culture and transfection

HeLa cells were grown in Dulbecco’s modified Eagle medium (Invitrogen, Carlsbad, CA, USA), supplemented with 10% fetal bovine serum and 1% glutamine. For transfection of siRNA, 125 pmol of custom siRNAs were transfected with Lipofectamine 2000 (Invitrogen, Carlsbad, CA, USA), according to the manufacturer’s instructions. siRNA sequences used were: human FUS/TLS: 5^′^-GGCCAAGAUCAAUCCUCCAUGAGUA-3^′^, and #2 5^′^-CGGGACAGCCCAUGAUUAAUUUGUA-3^′^; human G3BP1: 5^′^-ACAUUUAGAGGAGCCUGUUGCUGAA-3^′^, and #2 5^′^-GCGCAUUAACAGUGGUGGGAAAUUA-3^′^; human TDP-43: 5^′^-AAGCAAAGCCAAGAUGAGCCUUUGA-3^′^, and #2 5^′^- AAGAUGAGAACGAUGAGCCCAUUGA-3^′^, and negative control low GC siRNA (catalogue number 12935-200, Invitrogen, Carlsbad, CA, USA). Cells were transfected at 30–50% confluency and collected after 72 hr. For transfection of cDNA, 0.3 μg GFP tagged TIA-1 (provided by J. Goodier, Johns Hopkins University, MD) or 0.3 μg of G3BP-GFP (provided by J. Tazi, Montpellier, France) were transfected with Lipofectamine LTX (Invitrogen, Carlsbad, CA, USA) according to the manufacturer’s instructions. Cells were transfected with cDNA 48 hr after siRNA treatment. Cells were collected after 24 hr of treatment. Cells were treated with 0.5 mM sodium arsenite (30 min, 37°C; Sigma). Recovery was initiated by changing the media. Cells were fixed at the indicated time points.

### Immunofluorescence and antibodies

Cells grown on cover slips were fixed in 1% formaldehyde in phosphate buffered saline (PBS) and subsequently permeabilized with 0.1% Triton X-100 in PBS for 15 min. Cover slips were blocked with 0.1% bovine serum albumin (BSA) in PBS for 15 min and incubated with antibodies to TDP-43 (1:400; Proteintech, Chicago, IL, USA), TIA-1 (1:100; Santa Cruz, Santa Cruz, CA, USA), G3BP (1:400; BD Biosciences, Franklin Lakes, NJ, USA), diluted in blocking buffer for 1 hr at room temperature. Cover slips were subsequently washed once with 0.1% Triton X-100 in PBS and twice with 0.1% BSA in PBS. Labelling was visualized with the fluorescently conjugated secondary antibodies donkey anti-mouse Texas Red (1:200; Jackson Immunochemicals, West Grove, PA, USA), donkey anti-rabbit FITC (1:200; Jackson Immunochemicals, West Grove, PA, USA), and donkey anti-goat Alexa 633 (1:800; Invitrogen, Carlsbad, CA, USA). For some experiments, cells were stained with TO-PRO 3 (1:300; Invitrogen, Carlsbad, CA, USA) 10 min at room temperature. Cover slips were washed tree times in PBS and mounted with ProLong Antifade reagent (Invitrogen, Carlsbad, CA, USA). Images were collected on a Leica SP5 confocal microscope.

### Quantification of SG size and number

ImageJ was used for quantification of SG parameters. SGs were identified by TIA-1 staining and cells were scored as positive when they had at least two foci of a minimal size of 0.75 μm^2^. Automatic recognition was done by Image J using the following parameters, SGs ranging from 0.75 to 5 μm^2^, randomly selected in 10 cells per condition. The average SG size of at least 100 SG is presented. For G3BP-GFP experiments, cells with an average pixel intensity of 60 arbitrary fluorescent units were selected for analysis. Control cells at this level of G3BP-GFP expression did not exhibit SG formation in basal conditions.

### Cell lysates and immunoblot analysis

Cells were collected in ice-cold PBS, lysed in RIPA buffer (150 mM NaCl, 50 mM Tris pH 7.4, 1% Triton X-100, 0.1% SDS, 1% sodium deoxycholate, and protease inhibitors), incubated 10 min on ice, 10 min at room temperature, and centrifuged at 13 800 *g*. Supernatants were collected and quantified with the BCA Protein Assay Kit (Thermo Scientific, Waltham, MA, USA). The following antibodies were used in immunoblotting: rabbit anti-TDP-43 (1:5000; Proteintech, Chicago, IL, USA), goat anti-TIA-1 (1:500; Santa Cruz Biotech, Santa Cruz, CA, USA), mouse anti-G3BP (1:750; BD Biosciences, Franklin Lakes, NJ, USA), rabbit anti-FUS (1:1000, Abcam, Cambridge, MA, USA) and mouse anti-Actin (1:200 000; MP Biomedicals, Santa Ana, CA, USA). Blots were visualized with peroxidase-conjugated secondary antibodies and ECL Western Blotting Substrate (Pierce, Waltham, MA, USA). Densitometry was performed with Adobe PhotoShop.

### Protease sensitivity

Cells were collected in ice-cold PBS, lysed in 10 mM Tris pH 8.0, 50 mM NaCl, 0.5 M sucrose, 0.1 mM EDTA, 0.5% Triton X-100, 1 mM DTT. Cytoplasmic fractions were recovered as supernatants after centrifugation at 230 *g* for 10 min and quantified with the BCA Protein Assay Kit (Thermo Scientific, Waltham, MA, USA). 50 μg of cytoplasmic extract were incubated with increasing concentrations of Proteinase K (0 - 1.6 mg/ml) for 5 min at 37°C. Proteinase K was neutralised by 10 mM PMSF for 10 min on ice [36]. Samples were then resuspended in 1X Laemmli sample buffer.

### Cell viability

Cell viability in siRNA depleted cells was scored via trypan blue exclusion at the indicated times after stress. Cell viability/toxicity in GFP-TIA-1 transfected cells was measured using the Annexin V/7-AAD labelling kit (BD Biosciences, Franklin Lakes, NJ, USA) according to the manufacturer’s instructions In these experiments, cells were gated for GFP and Annexin V^+^ cells are reported in the figure. Data acquisition was performed on a BD LSR II flow cytometry (BD Biosciences) and data was analyzed with FlowJo (Treestar, Ashland, OR).

### Statistics

In all cases, data was compared via a two-tailed Student t-test using Microsoft Excel.

## Abbreviations

ALS: Amytrophic Lateral Sclerosis; FUS/TLS: Fused in Sarcoma/Translocated in Sarcoma; G3BP: RasGAP SH3 domain binding protein 1; HDAC6: Histone Deacetylase 6; SA: Sodium Arsenite; SG: Stress Granule; SMA: Spinal Muscular Atrophy; SMN: Survival Motor Neuron; TDP-43: TAR DNA response element binding protein 43; TIA-1: T-cell Intracellular Antigen.

## Competing interests

The authors declare they have no competing interests.

## Authors' contributions

AA and CVV designed experimental procedures. AA and SS performed the experiments. AA and CVV wrote the manuscript. All authors read and approved the final manuscript.

## Supplementary Material

Additional file 1 **Figure S1.** Blocked stress granule assembly confirmed with a second set of gene-specific siRNAs. HeLa cells were transfected with independent siRNAs for the indicated genes. SG number and size were quantified at the indicated times, as previously described in Figures 1, 2, 3 and 4. The means of 3 independent experiments ± SEM are plotted. * p < 0.05. Click here for file

Additional file 2 **Figure S2.** Stress granule initiation is not affected by G3BP and FUS. SG nucleation, as determined by TIA-1 labelling, was assessed in HeLa cells transfected with control, G3BP or FUS siRNA for 72 hr and subsequently treated with SA (0.5 m M, 30 min) and then immediately fixed. SG number and size were quantified using ImageJ. The means of 3 independent experiments ± SEM are plotted. * p < 0.05. Click here for file

Additional file 3 **Figure S3.** Toxicity is equivalent in high and low expressing GFP-TIA-1 transfectants**. (A–B)** HeLa cells transfected with GFP or GFP-TIA-1 for 48 hr (from Figure 6) were electronically gated for low and high TIA-1 expression. Cell viability, as determined by Annexin V labelling, was independently assessed in the two populations. The means of 3 independent experiments ± SEM are plotted. * p < 0.05. Click here for file
