# Erratum to: Endogenous TDP-43, but not FUS, contributes to stress granule assembly via G3BP

**DOI:** 10.1186/s13024-015-0041-8

**Published:** 2015-09-03

**Authors:** Anaïs Aulas, Stéphanie Stabile, Christine Vande Velde

**Affiliations:** Centre d’excellence en neuromique de l’Université de Montréal, Centre de recherche du Centre Hospitalier de l’Université de Montréal (CRCHUM), Departments of Medicine and Biochemistry, Université de Montréal, 1560 rue Sherbrooke Est, Montréal, QC H2L 4M1 Canada; CHUM Research Center (CRCHUM), Université de Montréal, 1560 rue Sherbrooke Est, Montréal, H2L 4M1 Canada

## Erratum

Since publication of our article [1] we have noticed that an error was introduced during the assembly of Fig. 1d (Fig. 1 here), resulting in the duplication of two panels. Specifically, lanes 7–12 probed for TIA-1 are the same as lanes 13–18 also probed for TIA-1. In addition, lanes 7–12 probed with Actin are the same as lanes 19–24 probed with Actin [1]. We sincerely regret this error. The corrected figure appears below.

**Fig. 1:**
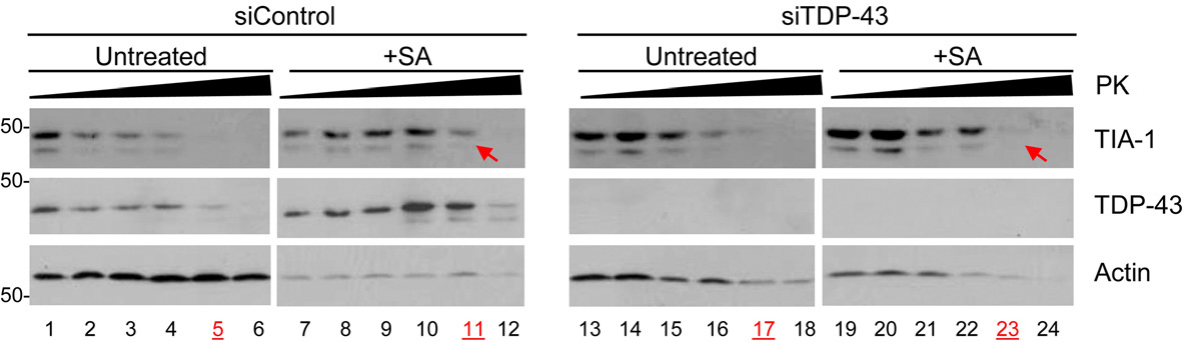
siRNA transfected HeLa cells were treated with or without SA and collected 1 h post-SA. Cytoplasmic extracts were digested with 0, 0.1, 0.2, 0.4, 0.8 or 1.6 mg/ml Proteinase K and assayed by immunoblot. TIA-1 is more protease-sensitive when TDP-43 is absent (arrows). Data is representative of 3 independent experiments
