# Supplementary figures and images for: Endogenous TDP-43, but not FUS, contributes to stress granule assembly via G3BP
Source: Mol Neurodegener. 2012 Oct 24;7:54. doi: 10.1186/1750-1326-7-54 (PMC3502460; doi:10.1186/1750-1326-7-54)

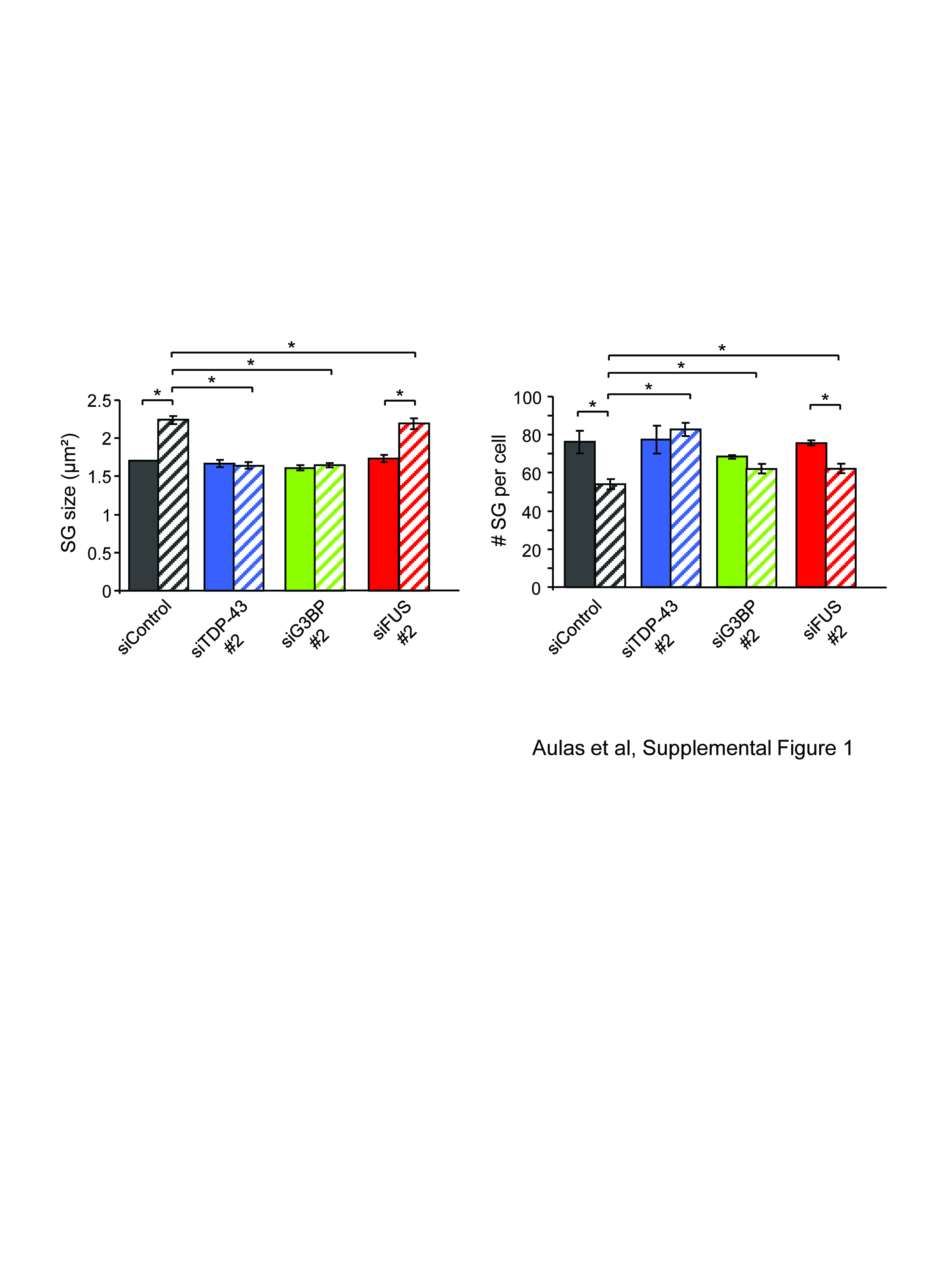

Supplement: Additional file 1 — Figure S1. Blocked stress granule assembly confirmed with a second set of gene-specific siRNAs. HeLa cells were transfected with independent siRNAs for the indicated genes. SG number and size were quantified at the indicated times, as previously described in Figures 1, 2, 3 and 4. The means of 3 independent experiments ± SEM are plotted. * p < 0.05. [file 1750-1326-7-54-S1.tiff]

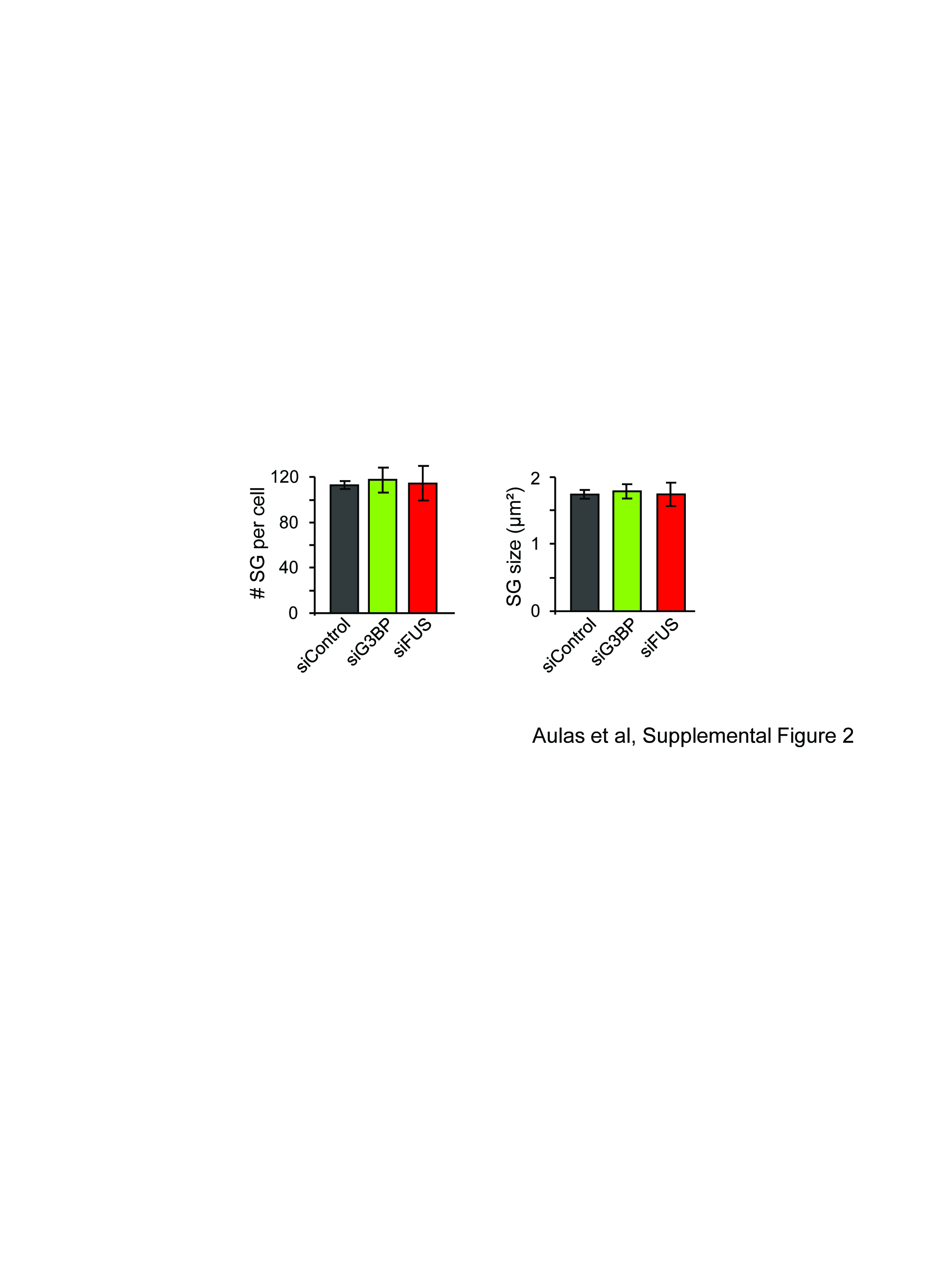

Supplement: Additional file 2 — Figure S2. Stress granule initiation is not affected by G3BP and FUS. SG nucleation, as determined by TIA-1 labelling, was assessed in HeLa cells transfected with control, G3BP or FUS siRNA for 72 hr and subsequently treated with SA (0.5 m M, 30 min) and then immediately fixed. SG number and size were quantified using ImageJ. The means of 3 independent experiments ± SEM are plotted. * p < 0.05. [file 1750-1326-7-54-S2.tiff]

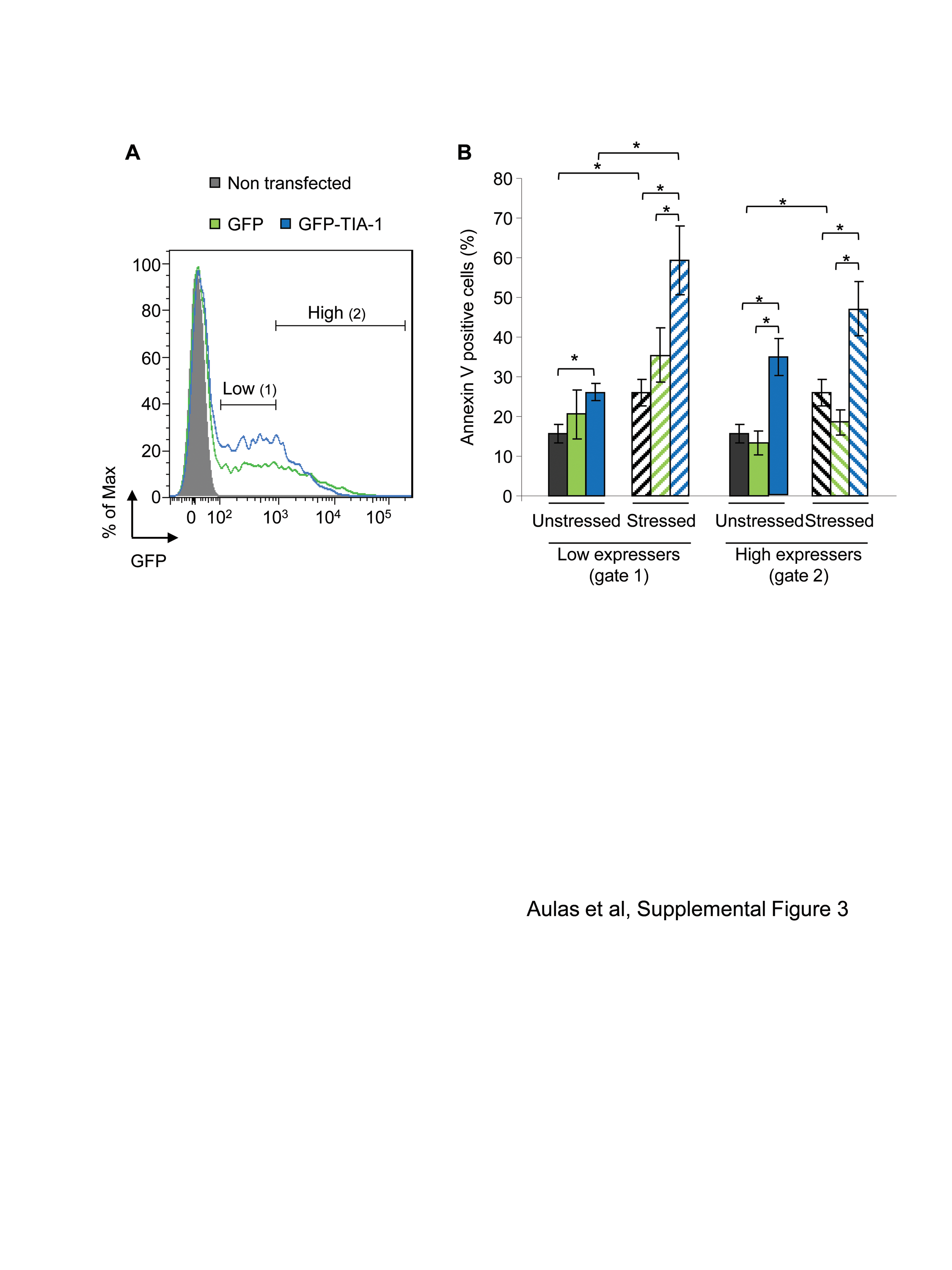

Supplement: Additional file 3 — Figure S3. Toxicity is equivalent in high and low expressing GFP-TIA-1 transfectants. (A–B) HeLa cells transfected with GFP or GFP-TIA-1 for 48 hr (from Figure 6) were electronically gated for low and high TIA-1 expression. Cell viability, as determined by Annexin V labelling, was independently assessed in the two populations. The means of 3 independent experiments ± SEM are plotted. * p < 0.05. [file 1750-1326-7-54-S3.tiff]
